# Supplementary material for: Multimodal phenotypic axes of Parkinson’s disease
Source: NPJ Parkinsons Dis. 2021 Jan 5;7:6. doi: 10.1038/s41531-020-00144-9 (PMC7785730; doi:10.1038/s41531-020-00144-9)
Supplement: Supplementary file 1 — Supplemental Information [file 41531_2020_144_MOESM1_ESM.pdf]

Multimodal phenotypic axes of Parkinson's disease

Ross D. Markello, Golia Shafiei, Christina Tremblay, Ronald B. Postuma, Alain Dagher, Bratislav Misic
McConnell Brain Imaging Centre, Montréal Neurological Institute, McGill University, Montréal, Canada

SUPPLEMENTARY RESULTS

SNF discriminates diagnostic groups

While the current report was primarily interested in investigating how SNF might serve to uncover putative biotypes of PD, we wanted to validate the use of SNF on identification of known diagnostic clusters in the same dataset. We ran the full SNF pipeline as described in the main text (see Fig. 1) including both healthy controls (n = 87) and PD patients (n = 186), to determine if SNF was able to correctly dissociate these groups. The resulting consensus cluster assignments yielded three clusters of n = 98, 84, and 91 individuals (Supplementary Fig. 1); the third cluster (n = 91 individuals) showed strong overlap with the healthy control population (F1 score = 0.94), while the other two clusters split the PD population (combining the two clusters yielded an F1 score of 0.97). Notably, the healthy control cluster is easily distinguishable from the PD patient clusters along the first dimension of the embedding space (Supplementary Fig. 1). This is quite distinct from the seemingly arbitrary groupings the PD biotypes make in the embedded space shown in Fig. 4, supporting the notion that biotypes may not be the most parsimonious characterization of PD (see *Discussion*).

Assessing longitudinal clinical outcomes

To better investigate the clinical utility of the biotypes described in the main text we compared longitudinal clinical outcomes for these biotypes with those derived from different subsets of the data and other clustering techniques. Though there are numerous clinical variables to examine, we chose to focus on the two highlighted in the main text: the tremor dominant score (tremor) and the postural instability/gait difficulty score (PIGD).

First, we examined whether biotypes obtained solely from baseline clinical assessments—excluding CSF assays, DAT scans, and neuroimaging data—showed differences in clinical outcomes over time for these two variables. Clustering labels were derived using the same SNF grid search pipeline described in *Results: Similarity network fusion provides a viable alternative to data concatenation*, but using only baseline clinical assessments as input. We observe trends suggesting that PIGD scores are discriminable between biotypes at baseline, and there are no changes in this discriminability over time (Supplementary Fig. 2b); tremor scores appear to be largely overlapping between biotypes.

Next, we examined whether biotypes derived from clustering using concatenated data showed discriminable clinical outcomes. These biotypes showed limited differences in PIGD and tremor scores at baseline, and relatively limited changes in discriminability over time (Fig. 2c). Interpretation of these results are supported by model estimates from linear mixed effects models run for both biotype definitions (Supplementary Table 5).

PCA is biased by data dimensionality

In the current article we compared clustering of PD patient data using SNF and data concatenation, showing that SNF better integrates data from different modalities and is less influenced by input data dimensionality. Here, we examine a similar comparison between the diffusion map embedding results presented in the main text and another low-dimensional projection technique: principal components analysis (PCA).

First, we computed the PCA of the concatenated patient by feature data matrix, yielding a vector of eigenvalues and corresponding eigenvectors. To investigate the similarities between this projection and the diffusion map embedding results we plotted the patient scores projected onto the first two principal components (PCs; Supplementary Fig. 3a). Examining these projections when colored by patient affiliation with the PD biotypes reported in the main text reveals a much less distinct separation between biotypes than is observed with the diffusion map embedding projection (Fig. 4b). We confirmed these differences between projections by correlating patient scores along the first two PCs with their complementary scores along the first two dimensions of the diffusion map embedding, yielding low—but nonzero—correlations (r_{PC1} = 0.142, r_{PC1} = 0.266; Supplementary Fig. 3b).

To investigate whether the PCA results were biased by data dimensionality we computed a PCA independently for each data modality and correlated patient scores along the first PC of these decompositions with the patient scores along the first PC of the concatenated data matrix (Supplementary Fig. 3c). Notably, PC scores from the concatenated data matrix are almost perfectly correlated with PC scores along the first dimension of the cortical thickness data (r = 0.99998), while showing negligible correlation with PC scores from the other data modalities (r_{avg} = 0.01673). These results suggest that, like clustering, PCA of the concatenated data matrix is biased by data dimensionality and unsuitable for investigations of datasets like those presented in the current study.

Stability of SNF

Despite extensive validation in the original article [8], its successful application to similar neuroimaging datasets [5, 7], and the exhaustive parameter search employed in the current article, we wanted to assess the stability of SNF as it applies to the current dataset. We first compare clustering assignments generated from SNF when varying the dimensionality of cortical thickness data (see *Data dimensionality variation*). We then compare patient networks generated from four hyperparameter combinations, examining differences in the resulting clustering solutions and diffusion dimensions (see *Hyperparameter variation*). Finally, we investigate how our choice of distance metric (i.e., squared Euclidean distance) impacts the patient networks and results reported in the main text (see *Alternative distance metrics*).

For all analyses, we calculated normalized mutual information scores (NMI) between different clustering solutions, and used Pearson correlations to compare the diffusion embedding dimensions derived from the fused netowkrs.

Hyperparameter variation

Using the results of the parameter search described in *Network creation and fusion* we selected four "stable" regions of parameter space and investigated the extent to which resulting clusters and embedded representations varied across these regions. For each of the four selected supra-threshold regions we calculated the center-of-mass and extracted the relevant (1) cluster labels and (2) network embeddings for the specified hyperparameter combinations (K = 22, 44, 58, 88; μ = 2.6, 4.4, 6.3, 7.3).

We computed NMI between all pairs of hyperparameter combinations separately for each cluster number (n = 2, 3, 4), finding considerable consistency amongst the three-cluster solutions (NMI$_{three}$ = 0.71 ± 0.08 [0.63–0.86]) but notably lower consistency for the other cluster numbers (NMI$_{two}$ = 0.49 ± 0.15 [0.27–0.66]; NMI$_{four}$ = 0.55 ± 0.32 [0.26–0.92]). Embedding dimensions were very consistent across all parameter combinations (average r = 0.97 ± 0.03 [0.91–1.00]).

Alternative distance metrics

In the main text we use a squared Euclidean distance function to generate patient similarity networks from the original data feature matrices. While there is precedent for this choice [5, 7, 8], we also wanted to investigate the impact of the chosen distance metric on our results. Thus, we repeated the analyses described in the main text using two alternative distance metrics—cityblock (or Manhattan) distance and cosine distance–finding that both clustering results (NMI$_{cityblock}$ = 0.74, NMI$_{cosine}$ = 0.68) and embedding dimensions (average $r_{cityblock}$ = 0.93 ± 0.10 [0.65–0.99], r_{cosine} = 0.87 ± 0.10 [0.70–0.96]) were highly consistent with squared Euclidean distance.

Comparing clustering techniques

Clinical clustering ("subtyping") of individuals with Parkinson's disease has received significant attention in recent years, especially since the release of the PPMI dataset [3, 4]. In the main text we demonstrated that inclusion of neuroimaging data significantly contributed to biotype discriminability using similarity network fusion. Here, we compare these findings to the clustering framework of one recent clustering solution using the same dataset.

Clinical guidelines (Fereshtehnejad et al., 2017)

In 2017, Fereshtehnejad and colleagues [4] used hierarchical clustering of baseline clinical-behavioral assessments to find three subtypes of individuals with PD from the PPMI dataset. From this clustering procedure they generated a set of clinical guidelines for classifying patients into three groups. While the hierarchical clustering and clinical guidelines result in slightly different subgroup definitions, the authors contend that their guidelines are more robust to different data acquisition schemes. Indeed, these guidelines have already been successfully applied to other datasets [2] with replication of primary clinical group differences. Although the replications studies report limited differences on autopsy parameters between groups, they attribute this to a ceiling effect driven by the advanced stage of the disease at death.

We apply these clinical guidelines to cluster our patient sample and find a similar distribution of assignments to those reported in [4] (90 subjects classified as "mild motor-predominant", 68 as "intermediate", and 28 as "diffuse malignant"). Notably, these assignments have limited overlap with the SNF-derived clustering assignments reported in the main text (normalized mutual information = 0.026). Using univariate one-way ANOVAs we find patients grouped according to the criteria of Fereshtehnejad are only discriminable in the clinical-behavioral assessments on which the clusters were initially defined (seven features in total; FDR-corrected, q < 0.05). That is, we fail to observe any cluster differences in DAT binding, CSF assays, cortical thickness, or subcortical volume measurements; we also fail to observe cluster differences in the PD-ICA atrophy scores derived from [9] (see *Materials and Methods: PD-ICA Atrophy Calculation*).

[1] Cammoun, L., Gigandet, X., Meskaldji, D., Thiran, J. P., Sporns, O., Do, K. Q., Maeder, P., Meuli, R., and Hagmann, P. (2012). Mapping the human connectome at multiple

scales with diffusion spectrum MRI. *Journal of Neuroscience Methods*, 203(2):386–397.

[2] De Pablo-Fernández, E., Lees, A. J., Holton, J. L., and Warner, T. T. (2019). Prognosis and neuropathologic correlation of clinical subtypes of Parkinson disease. *JAMA Neurology*, 76(4):470–479.

[3] Faghri, F., Hashemi, S. H., Leonard, H., Scholz, S. W., Campbell, R. H., Nalls, M. A., and Singleton, A. B. (2018). Predicting onset, progression, and clinical subtypes of Parkinson disease using machine learning. *bioRxiv*.

[4] Fereshtehnejad, S.-M., Zeighami, Y., Dagher, A., and Postuma, R. B. (2017). Clinical criteria for subtyping Parkinson's disease: biomarkers and longitudinal progression. *Brain*, 140(7):1959–1976.

[5] Jacobs, G. R., Voineskos, A. N., Hawco, C., Stefanik, L., Forde, N. J., Dickie, E., Lai, M.-C., Szatmari, P., Schachar, R. J., Crosbie, J., Arnold, P. D., Goldenberg, A., Erdman, L., Lerch, J. P., Anagnostou, E., and H, A. S. (2020). Integration of brain and behavior measures for identification of data-driven groups cutting across children with asd, adhd, or ocd. *bioRxiv*.

[6] Seabold, S. and Perktold, J. (2010). Statsmodels: Econometric and statistical modeling with python. In *9th Python in Science Conference*.

[7] Stefanik, L., Erdman, L., Ameis, S. H., Foussias, G., Mulsant, B. H., Behdinan, T., Goldenberg, A., O'Donnell, L. J., and Voineskos, A. N. (2018). Brain-behavior participant similarity networks among youth and emerging adults with schizophrenia spectrum, autism spectrum, or bipolar disorder and matched controls. *Neuropsychopharmacology*, 43(5):1180.

[8] Wang, B., Mezlini, A. M., Demir, F., Fiume, M., Tu, Z., Brudno, M., Haibe-Kains, B., and Goldenberg, A. (2014). Similarity network fusion for aggregating data types on a genomic scale. *Nature Methods*, 11(3):333.

[9] Zeighami, Y., Ulla, M., Iturria-Medina, Y., Dadar, M., Zhang, Y., Larcher, K. M.-H., Fonov, V., Evans, A. C., Collins, D. L., and Dagher, A. (2015). Network structure of brain atrophy in de novo Parkinson's disease. *Elife*, 4:e08440.

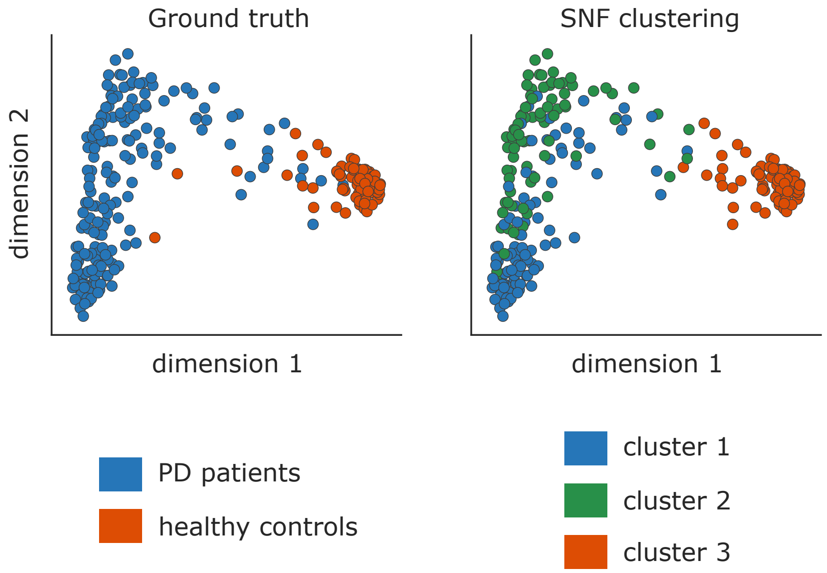

Supplementary Figure 1. **Similarity network fusion discriminates PD patients and healthy controls |** True diagnostic groups (left panel; PD patients and healthy controls) compared with SNF-derived clustering assignments (right panel), plotted along the first two dimensions of the embedding space derived from SNF. Healthy individuals are largely assigned to their own cluster by SNF (cluster 3, orange), whereas PD patients are split amongst two clusters (cluster 1, blue, and cluster 2, green).

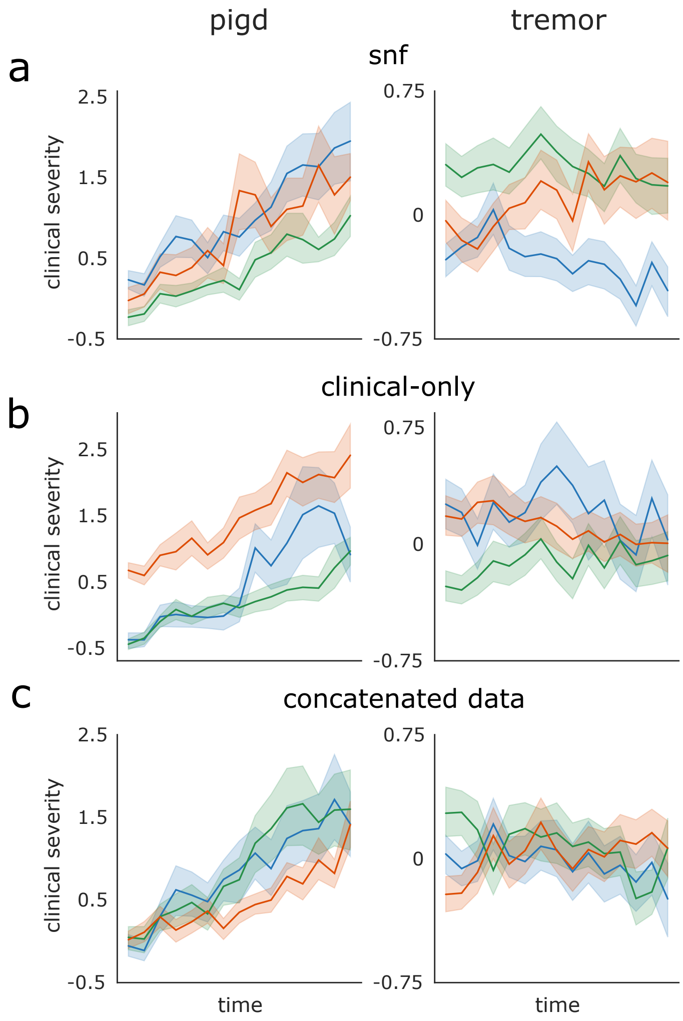

Supplementary Figure 2. **Longitudinal clinical outcomes vary based on data and clustering method | (a)** Reproduction of panel (e) from Fig. 3 showing longitudinal trajectories of SNF-derived biotypes for postural instability/gait disorder (PIGD) and tremor scores. **(b)** Longitudinal trajectories for biotypes derived from baseline clinical assessments only. **(c)** Longitudinal trajectories for biotypes derived from concatenated data.

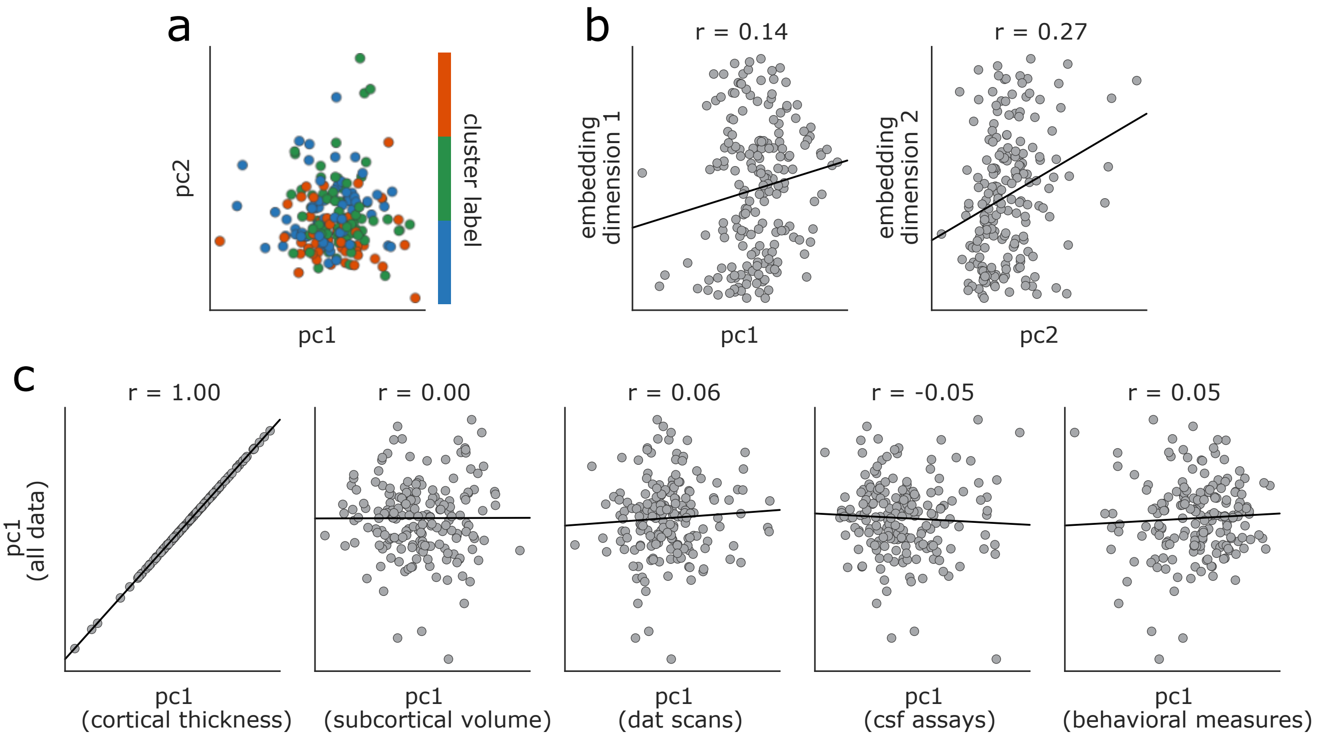

Supplementary Figure 3. **Comparison of low-dimensional embedding for SNF and data concatenation | (a)** Projection of PD patients onto the first two principal components derived from the concatenated data matrix. Patients are colored by their affiliation to the biotypes presented in the main text. **(b)** Correlations of patient scores along the first two principal components with corresponding scores along the first two diffusion map embedding dimensions. **(c)** Correlations of patient scores along the first principal component from the concatenated data matrix with corresponding scores along the first principal component of each independent data modality.

Supplementary Table 1. **Cluster demographics |** Demographic information on clusters defined in *Results: Derived patient biotypes are clinically discriminable across modalities*. Presented values are means ± standard deviations unless otherwise noted.

Cluster	Age (yrs)	Male (%)	White (%)	Education (yrs)
1	60.24 ± 8.62	51.39	91.67	15.47 ± 2.78
2	62.29 ± 10.17	60.87	92.75	15.81 ± 2.81
3	62.69 ± 8.83	64.44	88.89	15.56 ± 3.11

Cluster	Symptom duration (mo)	Family history of PD (%)	UPDRS total
1	6.21 ± 6.41	18.06	34.04 ± 13.35
2	5.74 ± 5.45	31.88	31.09 ± 13.37
3	5.56 ± 6.28	22.22	29.93 ± 11.66

Supplementary Table 2. **Data features supplied to SNF |** Cortical thickness features are not listed here due to their size, but can be found in a machine readable format on https://github.com/netneurolab/markello_ppmisnf. Designations of (+) and (–) indicate approximate phenotypic severity, where (+) denotes that higher scores for the relevant feature are considered "more severe" and (–) denotes that lower scores for the relevant feature are considered "more severe". A designation of (na) indicates that the relevant feature does not necessarily have a direct relationship to phenotypic severity. For all cortical thickness, subcortical volume, and DAT binding features lower scores are considered "more severe".

**Subcortical volume (–)**	**DAT binding (–)**	**CSF assays**	**Clinical assessments**
Caudate nucleus	Left caudate	Aβ (1-42) (–)	Benton (–)
Extended amygdala	Right caudate	CSF α-synuclein (–)	Epworth (+)
Globus pallidus, external	Left putamen	mtDNA deletion (+)	GDS (+)
Globus pallidus, internal	Right putamen	MT-ND1 copy number (+)	HVLT recall (–)
Habenular nuclei		MT-ND4 copy number (+)	HVLT recognition (–)
Hypothalamus		Phosphorylated tau (–)	HVLT retention (–)
Mammillary nucleus		Serum IGF-1 (–)	LNS (–)
Nucleus accumbens		Total tau (–)	MoCA (–)
Parabrachial pigmented nucleus			PIGD (+)
Putamen			QUIP (+)
Red nucleus			RBD (+)
Substantia nigra, pars compacta			SCOPA-AUT (+)
Substantia nigra, pars reticulata			Semantic fluency (–)
Subthalamic nucleus			STAI state (na)
Ventral pallidum			STAI trait (na)
Ventral tegmental area			Symbol digit (–)
			Systolic BP drop (+)
			Tremor (+)
			UPDRS-I (+)
			UPDRS-II (+)
			UPDRS-III (+)
			UPSIT (–)

Supplementary Table 3. **Data features discriminable between SNF-derived patient clusters |** Only features significant after FDR-correction ($q < 0.05$) are reported; note that no clinical-behavioral assessments survived this threshold. Numbers in parentheses next to cortical thickness features indicate regional sub-divisions; for more information on the parcellation refer to [1].

**Cortical thickness**	**Subcortical volume**	**DAT binding**	**CSF assays**
Right supramarginal gyrus (15)	Substantia nigra, pars compacta	Left caudate	Total tau
Right superior frontal gyrus (13)	Red nucleus	Left putamen	Phosphorylated tau
Right lateral orbitofrontal cortex (14)	Subthalamic nucleus	Right caudate	CSF α-synuclein
Right isthmus cingulate (5)	Parabrachial pigmented nucleus	Right putamen	
Right medial orbitofrontal cortex (10)	Ventral pallidum		
Right insular cortex (14)	Globus pallidus, internal		
Right superior frontal gyrus (20)	Substantia nigra, pars reticulata		
Left paracentral lobule (10)	Hypothalamus		
Left superior temporal gyrus (25)	Extended amygdala		
Left insular cortex (6)	Globus pallidus, external		
	Ventral tegmental area		
	Nucleus accumbens		
	Putamen		
	Habenular nuclei		

Supplementary Table 4. **Linear mixed effect model of tremor and PIGD scores |** Parameter estimates for two linear mixed effect models fit to longitudinal patient data with formula: `score ~ time * biotype + age + education + sex` [6]. Relevant data are shown in Figure 3.

	**Tremor model**				
	Estimate	*SE*	*Z-stat*	*2.5% CI*	*97.5% CI*
Intercept	-1.212	1.000	-1.213	-3.171	0.747
Biotype [T.2]	0.792	0.276	2.866	0.251	1.334
Biotype [T.3]	0.086	0.313	0.276	-0.526	0.699
Sex [M-F]	0.170	0.238	0.717	-0.295	0.636
Time	-0.081	0.024	-3.391	-0.128	-0.034
Time:Biotype [T.2]	0.074	0.034	2.193	0.008	0.140
Time:Biotype [T.3]	0.177	0.039	4.504	0.100	0.253
Age	0.042	0.013	3.378	0.018	0.067
Education	0.064	0.041	1.580	-0.015	0.144

	**PIGD model**				
	Estimate	*SE*	*Z-stat*	*2.5% CI*	*97.5% CI*
Intercept	-0.581	0.474	-1.225	-1.509	0.348
Biotype [T.2]	-0.281	0.132	-2.127	-0.540	-0.022
Biotype [T.3]	-0.147	0.150	-0.984	-0.440	0.146
Sex [M-F]	-0.067	0.113	-0.591	-0.287	0.154
Time	0.166	0.013	12.937	0.141	0.191
Time:Biotype [T.2]	-0.046	0.018	-2.533	-0.081	-0.010
Time:Biotype [T.3]	0.017	0.021	0.827	-0.024	0.058
Age	0.023	0.006	3.879	0.011	0.035
Education	-0.006	0.019	0.770	-0.043	0.032

Supplementary Table 5. **Longitudinal clinical outcomes vary based on data and clustering method |** Parameter estimates for supplementary linear mixed effect models fit to longitudinal patient data with formula: `score ~ time * biotype + age + education + sex` [6]. Biotypes are derived from either baseline clinical data only ("clinical-only") or concatenated multimodal data ("concatenated data"). Relevant data are shown in Supplementary Figure 2b-c.

	**Tremor model**									
	Clinical-only					***Concatenated data***				
	Estimate	*SE*	*Z-stat*	*2.5% CI*	*97.5% CI*	*Estimate*	*SE*	*Z-stat*	*2.5% CI*	*97.5% CI*
Intercept	-0.702	1.094	-0.642	-2.846	1.442	-1.233	1.055	-1.169	-3.301	0.834
Biotype [T.2]	-0.615	0.346	-1.781	-1.293	0.062	0.391	0.324	1.209	-0.243	1.026
Biotype [T.3]	-0.082	0.348	-0.235	-0.764	0.601	-0.150	0.288	-0.520	-0.714	0.414
Sex [M-F]	0.205	0.246	0.834	-0.277	0.687	0.291	0.252	1.154	-0.203	0.784
Time	-0.032	0.038	-0.854	-0.106	0.042	-0.042	0.025	-1.676	-0.091	0.007
Time:Biotype [T.2]	0.072	0.043	1.661	-0.013	0.157	-0.040	0.038	-1.052	-0.116	0.035
Time:Biotype [T.3]	-0.039	0.045	-0.870	-0.127	0.049	0.103	0.034	2.983	0.035	0.170
Age	0.041	0.013	3.112	0.015	0.067	0.045	0.013	3.435	0.019	0.070
Education	0.074	0.042	1.769	-0.008	0.156	0.070	0.042	1.663	-0.012	0.152

	**PIGD model**									
	Clinical-only					***Concatenated data***				
	Estimate	*SE*	*Z-stat*	*2.5% CI*	*97.5% CI*	*Estimate*	*SE*	*Z-stat*	*2.5% CI*	*97.5% CI*
Intercept	-0.604	0.477	-1.264	-1.541	0.333	-0.484	0.494	-0.981	-1.452	0.484
Biotype [T.2]	0.116	0.153	0.756	-0.184	0.416	-0.047	0.153	-0.304	-0.346	0.253
Biotype [T.3]	0.582	0.155	3.768	0.279	0.885	-0.036	0.136	-0.266	-0.303	0.230
Sex [M-F]	-0.159	0.107	-1.482	-0.369	0.051	-0.061	0.118	-0.518	-0.292	0.170
Time	0.209	0.020	10.486	0.170	0.248	0.170	0.013	12.664	0.143	0.196
Time:Biotype [T.2]	-0.099	0.023	-4.317	-0.144	-0.054	0.024	0.020	1.177	-0.016	0.064
Time:Biotype [T.3]	-0.027	0.024	-1.111	-0.073	0.020	-0.061	0.018	-3.332	-0.097	-0.025
Age	0.016	0.006	2.792	0.005	0.028	0.021	0.006	3.449	0.009	0.033
Education	0.000	0.018	0.026	-0.035	0.036	-0.011	0.020	-0.546	-0.049	0.028
