# Supplementary material for: Multimodal phenotypic axes of Parkinson’s disease
Source: NPJ Parkinsons Dis. 2021 Jan 5;7:6. doi: 10.1038/s41531-020-00144-9 (PMC7785730; doi:10.1038/s41531-020-00144-9)
Supplement: Supplementary file 2 — Reporting Summary FLAT [file 41531_2020_144_MOESM2_ESM.pdf]

Corresponding author(s): Ross Markello & Bratislav Misic

Last updated by author(s): Nov 3, 2020

Reporting Summary

Nature Research wishes to improve the reproducibility of the work that we publish. This form provides structure for consistency and transparency in reporting. For further information on Nature Research policies, see our Editorial Policies and the Editorial Policy Checklist.

Statistics

For all statistical analyses, confirm that the following items are present in the figure legend, table legend, main text, or Methods section.

n/a	Confirmed	
☐	☒	The exact sample size (*n*) for each experimental group/condition, given as a discrete number and unit of measurement
☐	☒	A statement on whether measurements were taken from distinct samples or whether the same sample was measured repeatedly
☐	☒	The statistical test(s) used AND whether they are one- or two-sided<br>*Only common tests should be described solely by name; describe more complex techniques in the Methods section.*
☐	☒	A description of all covariates tested
☐	☒	A description of any assumptions or corrections, such as tests of normality and adjustment for multiple comparisons
☐	☒	A full description of the statistical parameters including central tendency (e.g. means) or other basic estimates (e.g. regression coefficient) AND variation (e.g. standard deviation) or associated estimates of uncertainty (e.g. confidence intervals)
☐	☒	For null hypothesis testing, the test statistic (e.g. *F*, *t*, *r*) with confidence intervals, effect sizes, degrees of freedom and *P* value noted<br>*Give P values as exact values whenever suitable.*
☒	☐	For Bayesian analysis, information on the choice of priors and Markov chain Monte Carlo settings
☒	☐	For hierarchical and complex designs, identification of the appropriate level for tests and full reporting of outcomes
☐	☒	Estimates of effect sizes (e.g. Cohen's *d*, Pearson's *r*), indicating how they were calculated

Our web collection on statistics for biologists contains articles on many of the points above.

Software and code

Policy information about availability of computer code

Data collection	Data used in this study were obtained from the Parkinson's Progression Markers Initiative (PPMI) database (https://www.ppmi-info.org), accessed in March of 2018.
Data analysis	All code used for data processing, analysis, and figure generation is available on GitHub (https://github.com/netneurolab/markello_ppmisnf). Analyses were conducted in Python 3.6.7; the exact computing environment used to perform all analyses is packaged into a Singularity container, available on OSF (https://osf.io/h6jwx/).

For manuscripts utilizing custom algorithms or software that are central to the research but not yet described in published literature, software must be made available to editors and reviewers. We strongly encourage code deposition in a community repository (e.g. GitHub). See the Nature Research guidelines for submitting code & software for further information.

Data

Policy information about availability of data

All manuscripts must include a data availability statement. This statement should provide the following information, where applicable:

- Accession codes, unique identifiers, or web links for publicly available datasets
- A list of figures that have associated raw data
- A description of any restrictions on data availability

Data used in this study were obtained from the Parkinson's Progression Markers Initiative (PPMI) database (https://www.ppmi-info.org), accessed in March of 2018. Processed data used in the reported analyses are available on GitHub via https://github.com/netneurolab/markello_ppmisnf or via Zenodo at https://doi.org/10.5281/zenodo.3731252.

Field-specific reporting

Please select the one below that is the best fit for your research. If you are not sure, read the appropriate sections before making your selection.

☒ Life sciences ☐ Behavioural & social sciences ☐ Ecological, evolutionary & environmental sciences

For a reference copy of the document with all sections, see nature.com/documents/nr-reporting-summary-flat.pdf

Life sciences study design

All studies must disclose on these points even when the disclosure is negative.

Sample size	No sample size calculations were performed for the current analysis as we relied on the one-of-a-kind, open-access PPMI database. The 273 subjects (87 healthy controls and 186 patients with Parkinson's disease) were those who met inclusion criteria (see below).
Data exclusions	Refer to https://www.ppmi-info.org/study-design/research-documents-and-sops/ for documentation regarding exclusion criteria for subject enrollment. Subjects were excluded from data analysis if they (1) did not have at least one T1-weighted MRI scan or (2) were missing >20% of features from any data modality.
Replication	Analyses were not replicated as the authors are unaware of other Parkinson's disease datasets that contain all modalities of data collected by the PPMI for a similarly-sized (or larger) cohort (i.e., structural MRI, dopamine active transporter scans, biospecimen assays from cerebrospinal fluid, and broad clinical-behavioral assessments).
Randomization	Patients were allocated into experimental groups based on PD diagnosis made during study enrollment. Refer to https://www.ppmi-info.org/study-design/research-documents-and-sops/ for documentation describing diagnostic procedures.
Blinding	Investigators were not blind to experimental group as the analyses were focused solely on one of the groups (i.e., PD patients).

Reporting for specific materials, systems and methods

We require information from authors about some types of materials, experimental systems and methods used in many studies. Here, indicate whether each material, system or method listed is relevant to your study. If you are not sure if a list item applies to your research, read the appropriate section before selecting a response.

Materials & experimental systems

n/a	Involved in the study	
☒	☐	Antibodies
☒	☐	Eukaryotic cell lines
☒	☐	Palaeontology and archaeology
☒	☐	Animals and other organisms
☐	☒	Human research participants
☒	☐	Clinical data
☒	☐	Dual use research of concern

Methods

n/a	Involved in the study	
☒	☐	ChIP-seq
☒	☐	Flow cytometry
☐	☒	MRI-based neuroimaging

Human research participants

Policy information about studies involving human research participants

Population characteristics	Healthy individuals with no history of neurological illness (n = 87; ages 30-83, 32F/55M) and individuals diagnosed with Parkinson's disease (n = 186; ages 36-82, 78F/108M).
Recruitment	Refer to https://www.ppmi-info.org/study-design/research-documents-and-sops/ for documentation describing recruitment of subjects for the PPMI.
Ethics oversight	Central ethics oversight for the PPMI was provided by UCSF and additional oversight by individual research sites participating in the PPMI.

Note that full information on the approval of the study protocol must also be provided in the manuscript.

Magnetic resonance imaging

Experimental design

Design type	No task was run during scanning as all MRI scans used were structural images.

Design specifications	Scan time varied based on imaging sequence (see Acquisition, below).
Behavioral performance measures	No behavioral measures were collected during scanning.

Acquisition

Imaging type(s)	Structural (T1-weighted, T2-weighted, proton-density, FLAIR)
Field strength	1.5T & 3T
Sequence & imaging parameters	Imaging sequence varied by site in the PPMI; for a full list of sites and sequences please refer to https://www.ppmi-info.org/.
Area of acquisition	Whole-brain scans were used.

Diffusion MRI ☐ Used ☒ Not used

Preprocessing

Preprocessing software	The Advanced Normalization Tools (ANTs) software v2.2.0 was used for all image processing.
Normalization	T1w images were combined with other available neuroimages (T2-weighted, proton-density, and FLAIR images) across all subject timepoints to create a temporally unbiased, subject-specific template. A standard template was non-linearly registered to the subject template and used to remove non-brain tissue. The remaining subject template brain tissue was segmented into six classes (cerebrospinal fluid, cortical gray matter, white matter, subcortical gray matter, brain stem, and cerebellum) using ANTs joint label fusion with a group of fifteen expertly annotated and labeled atlases from the OASIS dataset. Finally, the segmented subject template was used to performing the same nonlinear registration, brain extraction, and segmentation procedures on the T1w images from each timepoint.
Normalization template	We used the MNI-ICBM152 non-linear 2009c asymmetric template for normalization.
Noise and artifact removal	T1-weighted structural images were corrected for signal intensity non-uniformity with N4 bias correction.
Volume censoring	No volume censoring was performed; all data were anatomical.

Statistical modeling & inference

Model type and settings	Cortical thickness and subcortical nuclei volumes were estimated for each subject from their processed T1w brain images. Where applicable, subjects were modeled as random effects.
Effect(s) tested	One-way ANOVAs were used to examine differences in imaging-based features of interest between derived PD patient biotypes.

Specify type of analysis: ☐ Whole brain ☐ ROI-based ☒ Both

Anatomical location(s)	ROI-based analyses were conducted using the Lausanne anatomical atlas, which is a subdivision of the Desikan-Killiany atlas (Cammoun et al., 2012).
Statistic type for inference (See Eklund et al. 2016)	No voxel-wise or cluster corrections were performed as whole-brain images were used solely for computation of a single, summary-statistic PD atrophy score.
Correction	FDR corrections (Benjamini-Hochberg) were used where appropriate to correct for multiple comparisons.

Models & analysis

n/a	Involved in the study
☒	☐ Functional and/or effective connectivity
☒	☐ Graph analysis
☐	☒ Multivariate modeling or predictive analysis

Multivariate modeling and predictive analysis	No multivariate modeling was performed on the MRI data itself; however, the data were used in multivariate data fusion analyses (i.e., similarity network fusion).
